# Comparing quality of recovery and satisfaction between spinal anesthesia and nerve block in orthopedic below-knee surgery: A prospective controlled trial

**DOI:** 10.1097/MD.0000000000037573

**Published:** 2024-04-05

**Authors:** Seon Woo Yoo, Taehoon Kim, Jongmin Seo, Hyunji Oh, Jun Ho Lee

**Affiliations:** a Department of Anesthesiology and Pain Medicine, Jeonbuk National University Medical School and Hospital, Jeonju, South Korea; b Research Institute of Clinical Medicine of Jeonbuk National University–Biomedical Research Institute of Jeonbuk National University Hospital, Jeonju, South Korea.

**Keywords:** enhanced recovery after surgery, femoral nerve, nerve block, orthopedic procedures, patient satisfaction, sciatic nerve, spinal anesthesia, ultrasonography

## Abstract

**Background:**

Postoperative quality of recovery (QoR) and patient satisfaction have gained increasing significance in medical services. This study aimed to compare these 2 parameters between 2 types of regional anesthetics (spinal anesthesia and combined sciatic-femoral nerve block) in orthopedic lower knee surgery.

**Methods:**

A total of 101 patients were classified into 2 groups (combined sciatic-femoral nerve block, group N; spinal anesthesia, group S) according to patient preference. In group N, sciatic and femoral nerve blocks were performed on the popliteal and groin regions, respectively, under ultrasound guidance. Spinal anesthesia was performed in group S. The primary outcomes were QoR and patient satisfaction. QoR was measured using the Korean translation of the QoR-15K. Patient satisfaction was assessed using an 11-point Likert scale (0–10) and a dichotomous question addressing anesthesia preferences for future surgeries.

**Results:**

The physical independence of the postoperative QoR-15K was significantly higher in group N than in group S (14.2 vs 12.0, *P* = .04). On the 11-point Likert scale, group N scored 8.8, and group S scored 7.8 (*P* = .001). In the dichotomous question, 93.8% of the group N and 52.8% of the group S answered that they would like to choose the same anesthesia method for the next surgery (*P* < .001). In addition, fewer participants in group N complained of backache than those in group S, and the time to first urination after anesthesia was shorter in group N than in group S (*P* = .004, <.001, respectively).

**Conclusion:**

Combined sciatic-femoral nerve block may provide better physical independence and satisfaction than spinal anesthesia in orthopedic below-knee surgeries.

## 1. Introduction

Today, as medical needs are being met and patients’ rights are increasing, improving the quality of medical services has become a major concern in most developed countries. Even in the field of surgery, recovery after surgery traditionally refers to the absence of significant side effects such as infection, hemorrhage, pneumonia, pulmonary embolism, or cardiac problems. However, this definition has recently evolved to encompass a state of comfort without physiological symptoms, such as nausea and pain.^[1]^ Furthermore, the comprehensive concept of postoperative recovery refers to multifaceted recovery that restores control over multiple functional domains, including psychological, emotional, social, and economic aspects. Additionally, satisfaction with the medical services provided has emerged as an important factor in postoperative recovery.^[2–4]^

Assessment of postoperative recovery has also shifted from the healthcare provider to the patient’s perspective. In previous studies, evaluating anesthesia management, postoperative mortality, morbidity, and adverse effects were primary concerns. However, recent clinical studies evaluating the impact of anesthesia management on qualitative aspects such as functional recovery, early recovery, and satisfaction have been in the spotlight.^[5]^ Accordingly, efforts are being made to develop various evaluation indicators for quantification and standardization. Quality of recovery-40 (QoR-40) is a widely used index to evaluate postoperative recovery. As the QoR-40 encompasses many questions and has been considered time-consuming, a simplified index, the QoR-15, was developed. The Korean translations of QoR-15 and 40, namely QoR-15K and 40K, have recently been validated, making them suitable for clinical use.^[6,7]^

Postoperative recovery is influenced by various factors such as the type of anesthesia, choice of anesthetic agent, analgesics or interventional procedures for pain, and integrated services after surgery, including opportunities for adequate communication with doctors and psychological support from medical staff. Among these, the type of anesthesia directly affects postoperative recovery. Most studies have compared various aspects of general anesthesia and regional anesthesia (neuraxial anesthesia or peripheral nerve block).^[8–10]^ However, few studies have compared QoR and patient satisfaction between the 2 distinct types of regional anesthesia.

Hence, we aimed to compare the QoR and patient satisfaction between spinal anesthesia and peripheral nerve block in orthopedic below-knee surgeries.

## 2. Methods

### 2.1. Patients and ethics

This prospective, parallel-group, controlled trial was approved by the hospital ethics committee of Jeonbuk National University Hospital Institutional Review Board (registration number: CUH 2021-07-008) and registered with the Clinical Research Information Service (KCT0008083). This study was conducted in accordance with the ethical principles of medical research involving humans, outlined in the Declaration of Helsinki of the World Medical Association. All patients were informed of the study, and written informed consent was obtained from all participants.

This study included 118 adult patients aged 18 to 80 years who were scheduled to undergo elective orthopedic below-knee surgery under combined sciatic-femoral nerve block (CSFNB) or spinal anesthesia at a tertiary care hospital between October 2021 and June 2023. The exclusion criteria were as follows: patients seeking general anesthesia, surgical position other than supine, contraindications to injection (coagulopathy, allergy to local anesthetics), patients who were expected to be difficult to cooperate with (patients with intellectual disabilities, moderate to severe dementia, severe psychiatric diseases), pregnant or lactating women, surgery lasting or expected to last more than 2 hours, or patients refusing patient-controlled analgesia (PCA). The first registration date was October 1, 2021 (Registration Number 1).

### 2.2. Study protocol

This study was a nonrandomized, parallel, prospective controlled trial. A researcher visited the study participants the day before surgery to determine their eligibility and obtain informed consent. The researcher administered a questionnaire to the participants to complete the preoperative QoR-15 and classified them into 2 groups according to the type of anesthesia (spinal anesthesia group, group S; CSFNB administration, group N). The group classification was not randomized; the researchers detailed the characteristics and procedures of the 2 types of anesthesia, and the patients selected one type of anesthesia according to their preference.

**Figure 1.**
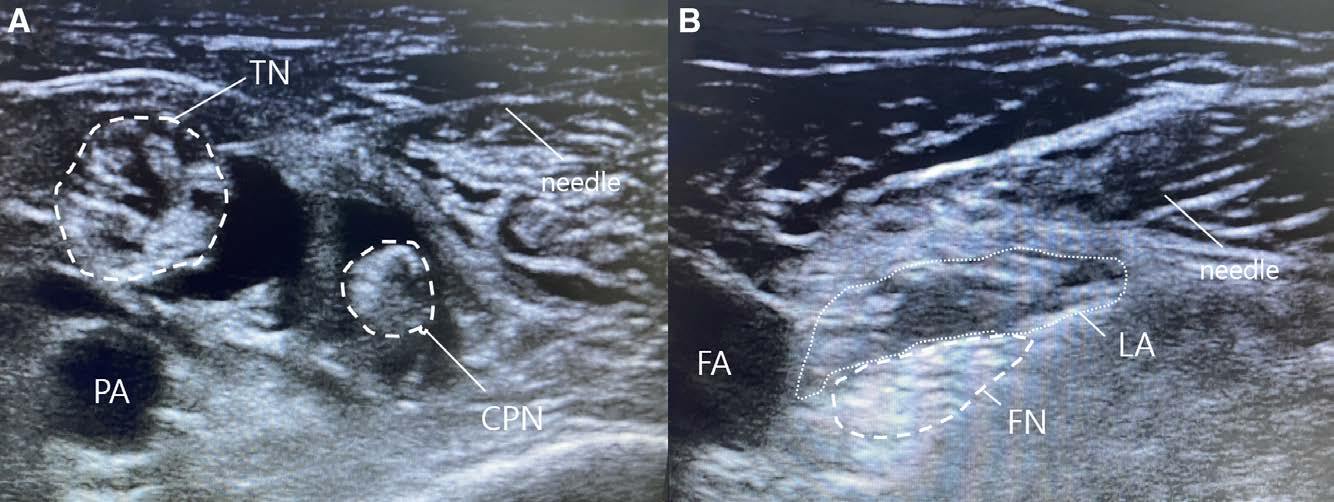
Ultrasound images of SN block (A) and FN block (B) in group N. (A) The TN and CPN are surrounded by LA in the area where the SN is bifurcated. (B) The FN is pushed downward as LA is injected under the fascia iliaca. CPN = common peroneal nerve, FA = femoral artery, FN = femoral nerve, LA = local anesthetics, PA = popliteal artery, SN = sciatic nerve, TN = tibial nerve.

After entering the operating room, all patients were routinely monitored using electrocardiography, noninvasive blood pressure measurements, and pulse oximetry. In group N, the sciatic and femoral nerves were injected once each for a total of 2 injections. The patient was first placed in the prone position, and under ultrasound guidance (high-frequency linear array transducer), the sciatic nerve was identified immediately before its bifurcation from the popliteal fossa to the common peroneal and tibial nerves, and a needle (26-gauge, 7 cm) was inserted laterally. Approximately 12 mL of 1.5% lidocaine mixed with epinephrine (0.1 mg) (1:1000 epinephrine) was injected through the paraneural sheath of the sciatic nerve.^[11,12]^ Anesthetic solution was injected by changing the angle of the needle surrounding the sciatic nerve. After the injection, ultrasound was performed to determine whether the hypoechoic fluid was sufficiently pooled around the sciatic nerve (Fig. 1A). After placing the patient in the supine position, the fascia iliaca was punctured under ultrasound guidance and approximately 12 mL of the same anesthetic solution was injected around the femoral nerve (the point where the deep femoral artery branches from the femoral artery in the groin area) (Fig. 1B). After approximately 5 minutes, a cold test was performed on the inside and outside of the calf and dorsum of the foot using an alcohol swab. If hypesthesia was incomplete, the cold test was repeated every 5 minutes. Intervention failure was determined if hypesthesia remained incomplete after approximately 20 minutes.

In group S, typical and standard spinal anesthesia was administered. First, the patient was placed in the lateral decubitus knee–chest position. A dura puncture was performed using a 25-gauge Quincke needle, and when clear cerebrospinal fluid (CSF) was observed, 10 to 15 mg of 0.5% hyperbaric bupivacaine (Marcaine-Heavy® 5 mg/mL, AstraZeneca, Seoul, Korea) was administered. The patient was placed in the supine position, and a cold test was performed to confirm whether anesthesia was adequately performed. In both groups, all procedures were performed by an experienced anesthesiologist (S.W.Y).

The heart rate (HR) and systolic blood pressure were recorded during surgery in all patients. All the patients received the same PCA dose for postoperative pain control. The regimen was as follows: total 100 mL, fentanyl 1000 mg, nefopam 120 mg, loading 0 mL, basal 0.1 mL/h, bolus 2 mL, and lock-out time 30 minutes. All patients were transferred to the postanesthesia care unit after surgery and discharged to the ward when their Aldrete score was 9 or higher.

## 3. Outcome measures

The primary outcomes of this study were the QoR-15K score and patient satisfaction. QoR-15K was surveyed twice (the day before surgery and 24 hours after surgery). Patient satisfaction was assessed 24 hours after surgery using an 11-point Likert scale (0–10) and a dichotomous question (whether you would choose the same type of anesthesia if you had the same operation the next time). The 11-point Likert scale was limited to satisfaction with anesthesia, with 0 being very dissatisfied and 10 being very satisfied. Secondary outcomes included intraoperative vital signs, postoperative PCA use, pain numerical rating scale score measured 24 hours after surgery, and side effects (nausea, vomiting, headache, dizziness, backache, local reaction at the injection site, and leg numbness). In addition, the time of urination, pain, and movement from the end of the surgery were recorded.

### 3.1. QoR-15K

The QoR-15K comprehensively evaluates physical comfort, emotional state, psychological support, physical independence, and physiological side effects such as pain. The QoR-15K comprises 15 questions, including 10 questions about emotions, and 5 questions about symptoms over the past 24 hours. Each question is rated on an 11-point Likert scale, with an overall score ranging from 0 to 150. The 15 questions evaluated 5 areas and consisted of 5, 4, 2, 2, and 2 questions on physical comfort, emotional state, psychological support, physical independence, and pain, respectively.

### 3.2. Statistical analysis

The sample size was calculated based on postoperative QoR-15K. An effect size of 0.57 was obtained for a minimal clinically important difference of 8.0 in a previous study.^[13]^ Considering a power of 0.8 and an allocation ratio of 1.2, a total sample size of 100 was obtained, and 118 patients were calculated, using G*Power 3.1, with a dropout rate of 15%. Data are expressed as mean ± standard deviation or median (25th–75th percentile), and number (%). *P* values <.05 were considered statistically significant.

Continuous variables, including QoR-15K, patient satisfaction, and PCA usage, were analyzed using Student *t* test or the Mann–Whitney *U* test after a normality test. Categorical variables, such as side effects, were analyzed using the Chi-square test or Fisher exact test. Hemodynamic parameters were compared using 2-way repeated-measures analysis of variance, and the Bonferroni *t* test was performed for post hoc analysis. All statistical analyses were performed using SPSS v27 (IBM Inc., Armonk, NY).

## 4. Results

**Figure 2.**
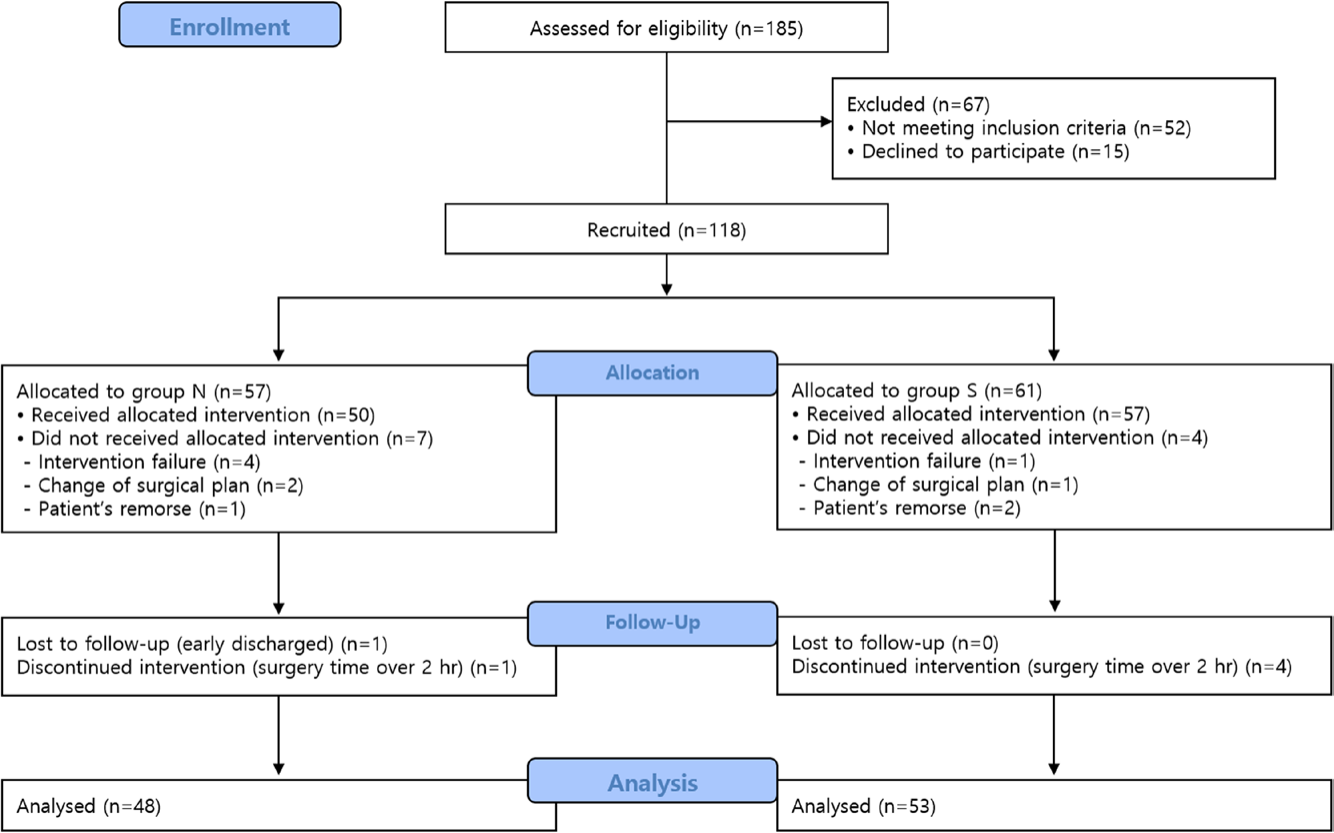
Consort-flow diagram.

The CONSORT flow diagram of this trial is shown in Figure 2. A total of 185 patients were screened for eligibility, and 118 were recruited and assigned to 2 groups according to the anesthetic method they voluntarily chose (group N = 57, group S = 61). Of these, the intervention failed in 5 patients, surgical plan was changed in 3 patients, and 3 wanted to change the anesthesia method to general anesthesia. One patient was lost to follow-up due to early discharge, and 5 patients were excluded from the analysis as the operation time exceeded 2 hours. Consequently, 101 patients (group N = 48, group S = 53) who completed the study were included in the analysis.

The baseline patient characteristics of the 2 groups were similar. There were no significant differences in the operation time or type of surgery (Table 1). There was also no significant difference in the total and 5 subcategories of the preoperative QoR-15K between the 2 groups (Table 2).

**Table 1:** Baseline patient characteristics.

	Group N (N = 48)	Group S (N = 53)	*P*-value
Age (yr)	58.4 ± 14.1	55.2 ± 16.6	.30
Sex (male/female)	27/21	26/27	.47
Height (cm)	161.8 ± 8.6	163.4 ± 9.5	.44
Weight (kg)	63.6 ± 8.6	67.4 ± 12.8	.08
BMI (kg/m^2^)	24.4 ± 3.5	25.3 ± 4.0	.26
ASA PS classification (I/II/III)	28/13/7	38/11/4	.14
Operation time, total (min)	38.6 ± 21.2	46.5 ± 26.0	.10
Type of surgery			.48
Fracture fixation	14	9	
Hardware removal	14	10	
Arthroscopic surgery	7	15	
Debridement	2	3	
Mass excision	1	3	
Deformity correction surgery	6	9	
Tendon/ligament repair	2	2	
Toe amputation	2	1	
Fascial graft	0	1	

Values are presented as the mean ± SD or median (25th–75th percentile) of the number of patients.

ASA-PS = American Society of Anesthesiologists physical status, BMI = body mass index, SD = standard deviation.

**Table 2:** QoR-15K scores.

	Group N (N = 48)	Group S (N = 53)	*P* value
Preoperative (range)			
Physical comfort (0–50)	47.1 ± 4.6	47.6 ± 4.1	.629
Emotional state (0–40)	34.9 ± 7.5	34.6 ± 7.4	.886
Psychological support (0–20)	19.0 ± 2.6	19.3 ± 1.4	.187
Physical independence (0–20)	16.6 ± 4.5	18.1 ± 3.4	.074
Pain (0–20)	15.1 ± 4.1	14.9 ± 5.6	.904
Total score (0–150)	132.6 ± 16.9	134.7 ± 16.7	.532
Postoperative (range)			
Physical comfort (0–50)	45.4 ± 6.1	42.6 ± 8.9	.069
Emotional state (0–40)	34.4 ± 7.5	32.5 ± 9.2	.270
Psychological support (0–20)	19.3 ± 1.9	18.8 ± 2.4	.235
Physical independence (0–20)	14.2 ± 5.6	12.0 ± 5.0	.038*
Pain (0–20)	13.5 ± 5.9	13.2 ± 5.8	.726
Total score (0–150)	125.7 ± 19.7	119.3 ± 25.2	.151

Values are presented as the mean ± SD.

QoR-15K = the Korean translation of the quality of recovery-15, SD = standard deviation.

* *P* < .05.

**Figure 3.**
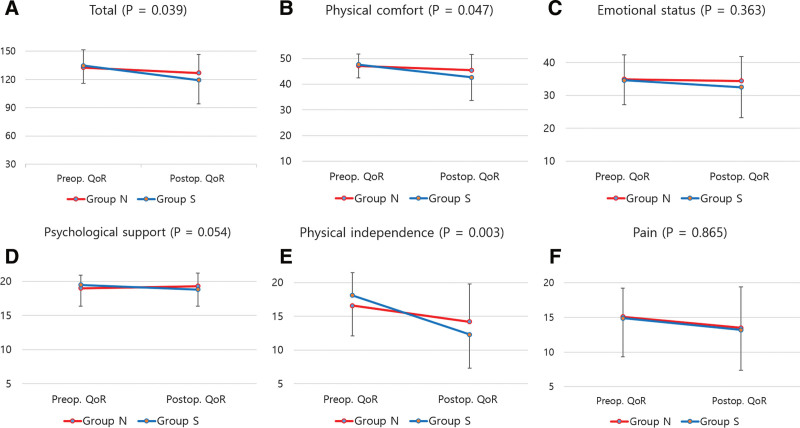
Preoperative and postoperative changes in QoR-15K. In group N, the total score of QoR-15K (A) decreased from 132.6 to 125.7; this decrease was significantly less than that in group S, which decreased from 134.7 to 119.3. In particular, there was a significant decrease in physical comfort (B) and independence (E). On the other hand, the changes in emotional status (C), psychological support (D), and pain (F) before and after surgery were similar in both groups. QoR = quality of recovery.

The total postoperative QoR-15K scores did not differ between the 2 groups. However, physical independence was significantly higher in group N than in group S (14.2 vs 12.0, *P* = .04). In comparing the difference in preoperative and postoperative QoR between the 2 groups, the decrease in the total score in group N was significantly smaller than that in group S (6.9 vs 15.4, *P* = .04, Fig. 3). In the subcategories, decreases in physical comfort and physical independence were significantly smaller in group N than in group S (1.8 vs 5.0, *P* = .05; 2.4 vs 6.0, *P* = .003, respectively). However, there was no significant difference in the degree of reduction between the 2 groups in terms of emotional status, psychological support, and pain.

On an 11-point Likert scale for satisfaction with anesthesia, group N scored an average of 8.8 points, and group S scored an average of 7.8 points. Regarding whether to select the same anesthesia for the next surgery, 45 of 48 patients in group N and 28 of 53 patients in group S answered yes. Both measures showed significant differences between the groups (*P* = .001, <.001, respectively).

**Figure 4.**
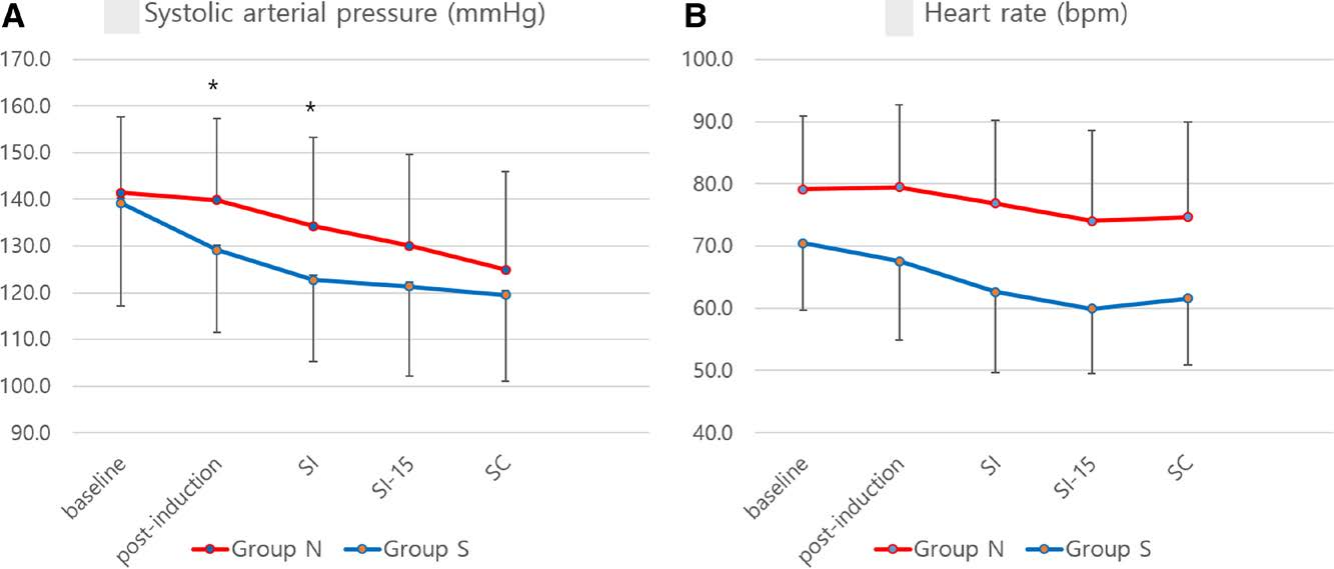
Changes in SBP and HR during surgery. There was a difference in the change over time between the 2 groups for both SAP and HR (*P* = .04 and .03, respectively) by 2-way repeated-measures analysis of variance. In group N, SAP was significantly higher at postinduction and skin incision than in group S (*P* = .003 and .002, respectively) by Bonferroni post hoc analysis. HR showed a difference between the 2 groups from baseline (*P* < .001). HR = heart rate, SAP = systolic arterial pressure, SC = skin closure, SI = skin incision, SI-15 = 15 min after skin incision.

Systolic artery pressure and HR (Fig. 4) showed changes over time, with significant differences between the 2 groups. In the Bonferroni post hoc test, systolic artery pressure was significantly lower in group S than in group N at the postinduction and skin incision points (*P* = .001 and < .001, respectively). In all patients, hemodynamic parameters were generally stable, and additional inotropes or vasopressors were not required.

PCA consumption and pain scores were similar in both the groups (Table 3). The time from the completion of surgery to urination was 265 minutes in Group N and 456 minutes in group S, showing a significant difference (*P* < .001), and the time to pain and movement were similar. Back pain was reported in 1 patient in group N and 11 patients in group S; there was a significant difference between the 2 groups (*P* = .004). However, nausea, vomiting, headache, dizziness, local reactions at the injection site, and leg numbness were similar. Leg numbness was reported in 8 patients; all cases were transient. The duration of postanesthesia care unit did not differ significantly between the 2 groups. There were no significant harmful or unintended effects in either group.

**Table 3:** Comparison of data between the 2 groups after surgery.

	Group N (N = 48)	Group S (N = 53)	*P* value
PCA consumption (mL)	27.2 ± 19.6	25.2 ± 19.4	.63
Pain score (NRS; 0–10)	3.3 ± 2.0	3.2 ± 1.9	.68
Time from end of surgery (min)			
Urination	265 ± 205	456 ± 224	<.001
Pain	346 ± 258	372 ± 240	.60
Movement	348 ± 203	385 ± 217	.39
Postoperative side effect (N [%])			
Nausea	13 (27.1)	16 (30.2)	.73
Vomiting	0 (0)	5 (9.4)	.06
Headache	1 (2.1)	6 (11.3)	.12
Dizziness	5 (10.4)	8 (15.1)	.48
Backache	1 (2.1)	11 (20.8)	.004
Local reaction at the injection site	0 (0)	3 (5.7)	.24
Leg numbness	4 (8.3)	4 (7.5)	>.99
Persistent nerve injury	0 (0)	0 (0)	>.99
Duration of PACU stay (min)	59.5 ± 22.1	61.2 ± 18.4	.68

Values are presented as the mean ± SD or the number of patients.

NRS = numerical rating scale, PACU = postanesthesia care unit, PCA = patient-controlled analgesia; SD = standard deviation.

## 5. Discussion

This study suggests that CSFNB results in better QoR than spinal anesthesia in patients undergoing orthopedic below-knee surgery. Although both groups experienced lower total postoperative QoR-15K scores than baseline, the decrease was more prominent in group S than in group N. In the subcategories, there was a significant difference in postoperative physical independence, which can easily be assumed because the area of anesthesia was relatively small in group N, resulting in better physical performance in the early stage of recovery. In addition, there was a significant difference in the reduction in physical comfort and physical independence. These differences are significant, particularly given that these anesthetic methods share the same main characteristics, whereby both modalities are administered via perineural injection.

Another notable finding of this study was the significant difference in patient satisfaction between the 2 groups. A high percentage (93.8%) of participants in group N expressed their preference to receive the same anesthesia in future procedures, with a satisfaction score of 8.8 on the Likert scale. Upon investigating the reason behind such a preference, the main factors were minimal injection pain, relatively less fear of anesthesia, quick recovery from anesthesia, less pain after surgery, and no major inconvenience. In addition, although this was not a crossover study, significant positive feedback was received from patients who previously received other types of anesthesia. On the other hand, the Likert scale score of group S was relatively low, at 7.8 points, and only 52.8% responded affirmatively to the question of whether to select the same anesthesia for future surgery. The contributing factors for negative answers included a certain period of forced bed rest, subsequent backache, slight chest tightness and nausea during surgery, numbness in both lower extremities, urinary disturbance, and gait delay.

Although somewhat controversial, it is generally accepted that regional anesthesia (neuraxial anesthesia and peripheral nerve block) has a slightly lower mortality rate, less postoperative delirium, and fewer analgesic requirements compared to general anesthesia. In particular, several studies focusing on elderly patients with hip fractures have reported that spinal anesthesia has better outcomes than general anesthesia in terms of mortality, delirium, intensive care unit admission, bleeding, and analgesia.^[14–16]^ Several authors have reported that spinal anesthesia provides a higher quality of initial recovery than general anesthesia.^[17–19]^ This superiority is related to physical comfort, reduced pain and nausea, increased mental independence, physical autonomy, maintenance of orientation, and emotional well-being. In particular, unpleasant feelings and sudden severe pain can lead to agitation, which is a disadvantage of general anesthesia compared to regional anesthesia.^[20]^ Therefore, patient satisfaction was generally higher or similar in patients who received regional anesthesia than in those who received general anesthesia.^[21–23]^

Regional anesthesia is classified into neuraxial and peripheral nerve blocks. Peripheral nerve anesthesia is the correct term; however, it is described as a peripheral nerve block, considering what has been commonly used. Comparisons were made with outpatient knee arthroscopies in a small number of studies that reported that CSFNB provides adequate anesthesia and patient satisfaction, similar to spinal anesthesia. CSFNB was reported to produce more favorable results for spontaneous urination, early postoperative pain, and preparation for discharge in outpatient knee arthroscopy.^[24–28]^ In a meta-analysis of outpatient surgery, peripheral nerve block had a potential advantage over neuraxial anesthesia in reducing the duration of stay in the recovery room and nausea after anesthesia.^[29]^ When comparing CSFNB and spinal anesthesia performed in elective below-knee surgery similar to this study, Pattajoshi et al^[30]^ found that CSFNB required longer anesthesia induction time than spinal anesthesia but showed superiority regarding initial analgesia, urination, and side effects (nausea/vomiting, hypotension). However, the authors did not draw any conclusions regarding QoR or patient satisfaction. In another study, Jeon et al^[31]^ reported that popliteal sciatic nerve block requires a longer procedure time than spinal anesthesia but provides fewer side effects, effective postoperative pain control, an appropriate level of anesthesia, and high patient satisfaction in hallux valgus surgery.

Regional anesthesia is introduced through a needle prick and by injecting drugs near a group of nerves or a single nerve to paralyze the body part. This procedure is one of the most critical factors affecting QoR and patient satisfaction. Notably, the proficiency of the anesthesiologist who performs the procedure determines the success of the process and the patient’s pain, discomfort, and anxiety.^[32,33]^ As ultrasound-guided nerve block requires both ultrasound technique and needle handling simultaneously, and detailed manipulation based on considerable experience is required. If the needle tip pierces the nerve and the needle is inserted intrafascicularly, even a small amount of solution can cause axonal degeneration and permanent neural damage.^[34–36]^ In this study, the procedure was performed by an experienced anesthesiologist who had performed ultrasound-guided injections for a considerable period of time. Most procedures were performed within 5 minutes, with no side effects such as nerve damage. This was considered one of the reasons for the high satisfaction in Group N.

Group N showed better results than group S in terms of urination time and backache. CSFNB has been suggested to improve urinary difficulty or retention, a common discomfort experienced after spinal anesthesia.^[37,38]^ In addition, nonspecific back pain caused by long-term immobility during surgery may be mistaken for back pain caused by spinal anesthesia. Notably, the use of the CSFNB eliminates these misunderstandings. Both groups were hemodynamically stable; however, the initial blood pressure decrease was significantly smaller in group N than in group S, which is considered to make CSFNB more advantageous. The HR findings were not meaningful, as the HR showed a difference between the groups at baseline.

This study is significant because it is the first to compare spinal anesthesia and CSFNB, focusing on QoR and patient satisfaction. However, this study has several limitations. First, randomization was not performed for the group classification. This is considered a major limitation of this study. However, we considered that patients’ preferences for the anesthesia method may affect QoR and patient satisfaction. Patients often chose the type of anesthesia they preferred based on their previous experience and knowledge, which was expected to clearly affect the results. For example, if a patient who strongly desired spinal anesthesia underwent CSFNB or vice versa, it was assumed that patient satisfaction or QoR would be lowered. Second, QoR-15K was used instead of QoR-40K, which is more precise and subdivided. However, in a previous study conducted in the same institution, we received feedback that QoR-40K increases the fatigue of the participants and may result in insincere answers due to the large number of questions. Repeating the same questionnaire 3 or more times can also cause these problems. This study was simplified by administering the questionnaire only twice: 1 day before surgery for the baseline and 24 hours after surgery.

In addition, incomplete anesthesia was observed in 4 patients in group N, despite the block being performed in the correct position under ultrasound guidance. If this is not due to anatomical variations of the sural nerve,^[39]^ insufficient local anesthetic penetration may be considered. Therefore, more in-depth research assessing the degree of needle access for anesthesia, the minimum dose of local anesthetic, and ultrasound imaging for solution accumulation is needed. Notably, future research is expected to yield promising outcomes by considering various surgeries.

In conclusion, this study suggests that CSFNB performed by an experienced anesthesiologist during orthopedic below-knee surgery provides better QoR and patient satisfaction than spinal anesthesia. This finding not only underscores the potential benefits of CSFNB, but also emphasizes the importance of tailoring anesthesia techniques to specific surgical contexts for optimized patient outcomes.

## Author contributions

**Conceptualization:** Jun Ho Lee.

**Data curation:** Hyunji Oh.

**Formal analysis:** Jongmin Seo.

**Funding acquisition:** Seon Woo Yoo.

**Investigation:** Seon Woo Yoo, Hyunji Oh.

**Methodology:** Seon Woo Yoo.

**Resources:** Hyunji Oh.

**Software:** Jongmin Seo.

**Supervision:** Jun Ho Lee.

**Validation:** Taehoon Kim, Jongmin Seo.

**Visualization:** Taehoon Kim.

**Writing—original draft:** Seon Woo Yoo.

**Writing—review & editing:** Jun Ho Lee.
